# Sperm Cell Capture Based on ABH Antigen Differences to Separate Two Men in Mixed Seminal Stains

**DOI:** 10.1155/2021/7269237

**Published:** 2021-11-27

**Authors:** Mao-ling Sun, Ji-long Zheng, Bao-jie Wang, Jun Yao

**Affiliations:** ^1^School of Forensic Medicine, China Medical University, Shenyang 110122, China; ^2^Department of Forensic Medicine, China Criminal Police University, No. 38, Tawan Street, Huanggu District, Shenyang, Liaoning 110854, China; ^3^Key Lab of Forensic Science, Ministry of Justice (Academy of Forensic Science), Shanghai 200061, China

## Abstract

Personal identification of two individuals in mixed semen samples in forensic DNA testing in general usually involves analysis using autosomal and Y chromosome short tandem repeats (STRs). Results may exclude unrelated donors but cannot identify individuals. In this study, sperm cell capture based on ABH antigen differences was used to obtain the cells with the single ABO blood type. Immunohistochemical staining using labeled anti-A, anti-B, and anti-H antibodies and the laser microdissection system can be used to enrich sperm with different ABO types in mixed seminal stains from two individuals. Then, PCR amplification and capillary electrophoresis were performed to genotype the STR loci. To some extent, after sperm cell capture based on ABH antigen differences, autosomal STR typing using enriched single blood group cells can be utilized to partially identify different individuals in a mixed seminal stain sample from two individuals.

## 1. Introduction

A mixed stain means that the specimen being analyzed was derived from two or more individuals. It is frequently obtained in criminal scenes. In sex crimes, a victim may be sexually abused by two or more men at the same time. Evidence obtained in such cases corresponds to the semen of male donors and a mixed stain including vaginal fluid from the female victim. To identify male suspects, differential extraction procedures are used to remove the female composition from the sample, followed by autosomal small tandem repeat (STR) typing. After analyzing the mixed allele peaks in the spectrum, individual genotypes can be inferred [1, 2]. Presently, several useful statistical methods are available, such as STRmix™ software and likelihood ratio statistic [3–5]. They can be used to interpret the mixed DNA samples from multiple contributors. For Y chromosome STR, its profiles are often used when the autosomal STR genotyping cannot generate the useful profiles. However, it plays a limited role in the mixed male DNA samples [6]. Due to paternal inheritance of the Y chromosome, men belonging to one agnate have the same Y chromosome STR type [7, 8]. In this case, STR results can exclude unrelated individuals but does not achieve the purpose of identification. Therefore, it is easier and more credible to obtain and inspect individual specimens rather than merged peaks [9]. Thus, in order to identify the suspect, an unambiguous genotype analysis requires successful separation of the offender's cells from the mixed stains.

Single-cell specimens can be obtained through laser-capture microdissection (LCM) technology [9]. It can isolate the specific cells in the native niche without the contamination of the surrounding areas [10]. However, the nuclear DNA of a single sperm cell is haploid and does not satisfy the quantity requirements for sensitivity detection using PCR. Additionally, it cannot provide the complete genetic information about individuals. Sorting and enriching sperm cells for individual characteristics are critical to solving individual identification issues in mixed seminal stains.

The sperm cell surface contains ABH antigens, which correspond to individual ABO blood types. ABH antigen expression in sperm cells is controlled by the ABH gene, which is dependent on secretor status [11]. Secretors, who have ABO antigens in their body secretions such as saliva and semen, have at least one functional Se allele, whereas nonsecretors, who fail to express ABO antigens in their secretions, are homozygous for the nonfunctional Se allele [11]. This is the basis of our study. According to differences in ABH antigens in sperm cells, cell capture technology can be applied to enrich sperm with the same ABO blood group. Subsequently, the personal identification can be achieved by autosomal STR typing. Our method may provide an effective selection for the single male STR profile in mixed seminal stains.

## 2. Materials and Methods

### 2.1. Sample Collection

The protocol was approved by the Ethics Committee of China Medical University. All the participants were included after providing the written informed consent.

In the Chinese Han population, the distributions of ABO phenotypes were as follows: A: 29.02%; B: 28.06%; O: 34.05%; and AB: 8.87% [12]. Thus, blood samples were collected from 50 unrelated healthy male individuals to detect the ABO type and secretor status according to the reported method [13]. Then, four secretor individuals with A, B, AB, and O blood groups were selected to obtain the semen samples. Six pairwise mixed samples (volume ratio: 1 vs. 1) were composed of four different blood group samples.

### 2.2. Preparation and Staining of Mixed Seminal Stain

Mixed seminal samples were washed and suspended in 0.9% NaCl to a density of 1 × 10^7^/ml using the cell counter (Countess 3, Thermo Fisher Scientific, Waltham, MA). The 20 *μ*l sperm suspension was smeared and blown under a thermal fan for 20 min. The mixed seminal stain was placed in 100% methanol for 30 min and washed with phosphate-buffered saline (PBS, pH 7.2) 2 times for 10 min each. Triton X-100 PBS (0.1% with 3% BSA) was used to seal the mixed seminal stain in a humidified box at room temperature for 1 hour. The mixed seminal stain was incubated in anti-A, anti-B, and anti-H antibodies labeled with three fluorescent colors (Shanghai Youke Biotechnology Co.) to stain overnight at 4°C. The antibodies were monoclonal antibodies and prepared in the mouse. Their specificity has been documented by the manufacturer. For the anti-A antibody, the fluorescent tag was NSH-fluorescein and the emission wavelength was 493 nm. For the anti-B antibody, the fluorescent tag was NSH-AMCA and the emission wavelength was 346 nm. For the anti-H antibody, the fluorescent tag was NSH-rhodamine and the emission wavelength was 555 nm. Under immunofluorescence detection, the colors that the A, B, and O antibodies presented were green, red, and blue, respectively. PBS without antibodies was used as a control.

### 2.3. Separation and Detection of Sperm Cells

A laser microdissection system (Leica LMD7000) was used to capture sperm positive for anti-A, anti-B, or only anti-H. The instrument fitted with a 355 nm UV-laser was used for laser microdissection of spermatozoa (400x magnification). A total of 50 cells were placed in a collection pipe, and DNA was extracted via the MagaZorb® DNA Mini-Prep Kit (Promega, Madison, WI, USA). The DNA quantification was performed using NanoDrop™ One Ultra-micro UV-Visible Spectrophotometer (Thermo Fisher). An autosomal STR kit (PowerPlex®16HS, Promega) was used to amplify 15 autosomal STR loci via PCR, according to the manufacturer's recommendations. One microliter of each PCR product was denatured in 10 *μ*l of loading buffer, composed of HI-DI™ formamide and Internal Lane Standard 600 (Promega) size standard mixture in a proportion of 500 : 1 (*v*/*v*). Separation and detection were performed on an Applied Biosystems™ 3130xl Series Genetic Analyzer™ (Thermo Fisher, Waltham, MA) using a 10 sec injection time and 3.0 kV injection voltage, previously described by Li et al. [14]. Raw data was analyzed using GeneMapper ID v3.2 software (Thermo Fisher). Internal controls (H_2_O as a negative and 9947A DNA as a positive control) were genotyped along with each batch of samples to ensure that the results were reproducible and accurate.

Finally, DNA extraction and autosomal STR genotyping were performed from four secretor individuals with A, B, AB, and O blood groups. Additionally, six pairs of mixed sperm samples of two individuals randomly combined were also genotyped using autosomal STR after separating and enriching by the laser microdissection.

## 3. Results and Discussion

**Figure 1:**
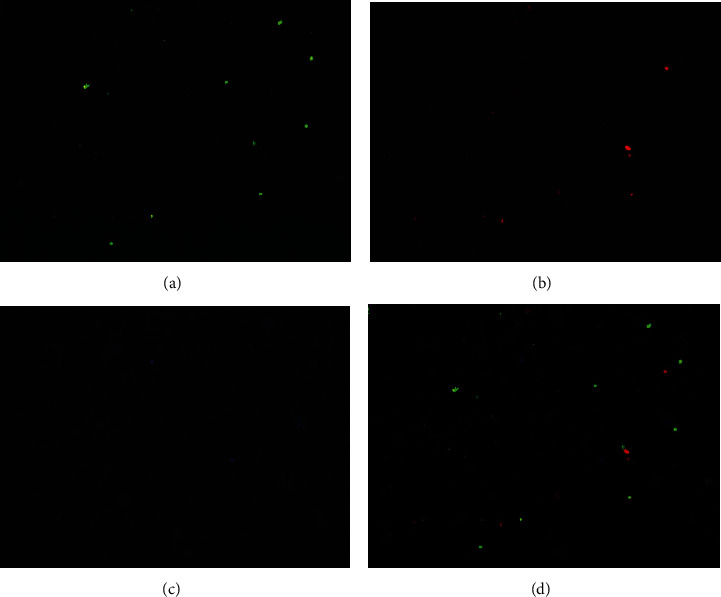
Immunofluorescence detection of AA and BO genotypes in a mixed sperm sample from two individuals: (a) positive sperm from type A; (b) positive sperm from type B; (c) positive sperm from type O; (d) superimposed type A, B, and O images.

**Figure 2:**
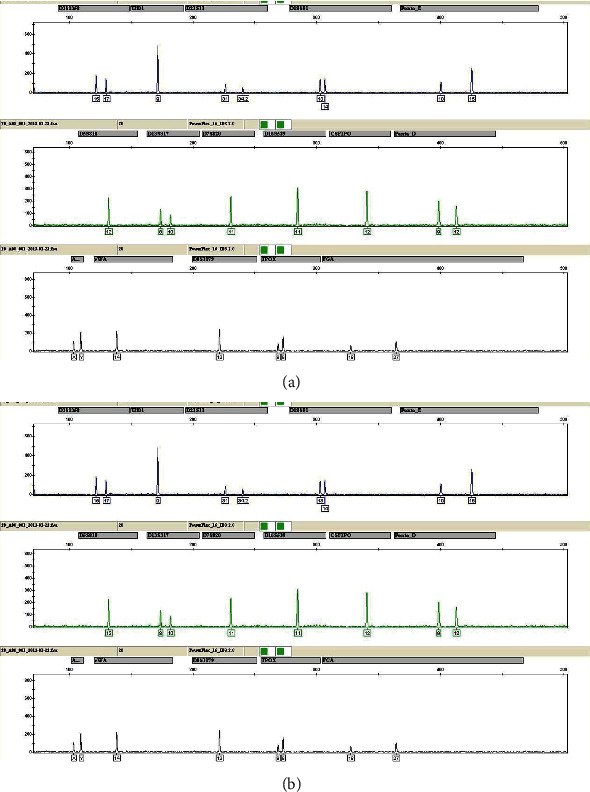
Autosomal STR genotyping cells removed by microdissection: (a) the genotype of the A blood-type sperm cell; (b) the genotype of the B blood-type sperm cell.

Immunofluorescence staining showed that A and B blood-type sperm cells were clearly differentiated (Figure 1). The anti-H antibody was weakly associated with the negative anti-A and anti-B types. Sperm that was positive for anti-H was from O blood-type individuals. For the mixed stains of A and B blood-type sperm cells, the autosomal STR typing for the two sperm cells collected was the same as the typing of the blood sample (Figure 2).

This test demonstrates that labeled anti-A, anti-B, and anti-H antibodies can be used to distinguish different sperm cells based on the ABO type. Due to differences in ABO types, the mixed sperm cells from two individuals can have 21 possible combinations (Table 1). Based on the discrimination probability of identifying the individuals in the mixed sample of two individuals, the classification of separation included determining two, one, or no individuals. Type I means that two individuals can be distinguished using this method. Type II means that one individual can be distinguished using this method. Type III means that this method cannot distinguish any individuals. For 9 genotype combinations of type I, such as AA/BB and AB/OO, this method can distinguish between two individuals with a proportion of 0.43 (9/21). For 6 genotype combinations of type II, such as AO/OO and AA/AB, this method can distinguish one of the two individuals with a proportion of 0.29 (6/21). For 6 genotype combinations of type III, such as AA/AA and BB/BB, this method cannot distinguish individuals, with a proportion of 0.29 (6/21). In Caucasians, the A, B, and O allele frequencies of the ABO blood group were 29.48%, 9.57%, and 60.95%, respectively. Thus, for type I combinations, the probability of distinguishing between two individuals was 27.93%. For type II combinations, the probability of distinguishing one of the two individuals was 42.91%. For type III combinations, the probability that this method cannot distinguish individuals was 29.16%. If only A, B, or O alleles are detected after ABO genotype detection in a mixed seminal stain from two individuals, this method cannot be utilized. However, the allele frequencies of the ABO blood group varied among the different populations. For example, in the Chinese Han population, the distributions of ABO phenotypes were as follows: A: 29.02%; B: 28.06%; O: 34.05%; and AB: 8.87% [12]. In the Mexican population, the distribution of ABO phenotypes was as follows: O: 61.82%; A: 27.44%; B: 8.93%; and AB: 1.81% [15]. Phenotypic frequencies of blood groups A, B, O, and AB were, respectively, 22.54%, 28.56%, 43.30%, and 5.60% in Burkina Faso [16]. Therefore, the discrimination capability of sperm cell capture based on ABH antigen differences is not the same in the different populations.

There were several limitations in the application of our method. Firstly, only the individuals with the sector status express the ABO blood antigen in the membrane of the sperm cells. Thus, this method cannot be used in the separation of the mixed stains from the nonsecretors. Secondly, it is unclear if the ABO subgroups, such as A2, Ax, and B3, will react with anti-A, anti-B, and anti-H antibodies and generate the same fluorescent intensity [17, 18]. This may give rise to the possibility of false negative. Finally, our method can be applied in the analysis of mixed stains only when individuals must have different ABO blood types and the mixed seminal stains should only be from two males. However, due to inherent limitations in the degree of polymorphism within the ABO blood system, this method cannot be used to differentiate all individuals. Additionally, if the suspects were from the same family group, the rapist cannot be identified by the Y-STR analysis due to the paternal inheritance of the Y chromosome.

In summary, sperm cell capture based on ABH antigen differences can be used to obtain the cells with the single ABO blood type. Immunohistochemical staining using labeled anti-A, anti-B, and anti-H antibodies and the laser microdissection system can be used to enrich sperm with different ABO types in mixed seminal stains from two individuals. Then, PCR amplification and capillary electrophoresis were performed to genotype the STR loci. To some extent, after sperm cell capture based on ABH antigen differences, autosomal STR typing using enriched single blood group cells can be utilized to partially identify different individuals in a mixed seminal stain sample from two individuals.

## Figures and Tables

**Table 1:** Different ABO genotype combinations in mixed sperm samples from two individuals.

Type I	Expected colors	Type II	Expected colors	Type III	Expected colors
AA/BB AA/BO AA/OO BB/AO BB/OO AB/AO AB/BO AB/OO AO/BO	Green/red Green/red Green/blue Red/green Red/blue Green/red Green/red Green/red Green/red	AA/AB AA/AO BB/AB BB/BO AO/OO BO/OO	Green/red Green/blue Green/red Red/blue Green/blue Red/blue	AA/AA BB/BB AB/AB AO/AO BO/BO OO/OO	Green Red Green/red Green/blue Red/blue Blue

Note: under immunofluorescence detection, the colors that the A, B, and O antibodies presented were green, red, and blue, respectively.

## Data Availability

The data used to support the findings of this study are included within the article.
